# Supplementary material for: The Synergistic Effect of Acidic Properties and Channel Systems of Zeolites on the Synthesis of Polyoxymethylene Dimethyl Ethers from Dimethoxymethane and Trioxymethylene
Source: Nanomaterials (Basel). 2019 Aug 23;9(9):1192. doi: 10.3390/nano9091192 (PMC6780150; doi:10.3390/nano9091192)
Supplement: Supplementary file 1 [file nanomaterials-09-01192-s001.zip › nanomaterials-562965-SI.pdf]

Supplementary Materials:

The Synergistic Effect of Acidic Properties and Channel Systems of Zeolites on the Synthesis of Polyoxymethylene Dimethyl Ethers from Dimethoxymethane and Trioxymethylene

Jianbing Wu [1,*], Sen Wang [2,*], Haitao Li [1], Yin Zhang [1], Ruiping Shi [2] and Yongxiang Zhao [1,*]

[1] Engineering Research Center of Ministry of Education for Fine Chemicals, Shanxi University, Taiyuan 030006, China

[2] State Key Laboratory of Coal Conversion, Institute of Coal Chemistry, Chinese Academy of Sciences, Taiyuan 030001, China

* Correspondence: wujianbing@sxu.edu.cn (J.W.); wangsen@sxicc.ac.cn (S.W.); yxzhao@sxu.edu.cn (Y.Z.)

1. More Experimental Details:

1.1. Catalyst preparation

SAPO-34 was synthesized with a gel composition of Al_2O_3:xP_2O_5:ySiO_2:3. 0Et_3N:zH_2O (x = 0.8~1.55, y = 0.1~0.85, z = 26~106) according to the literature [1]. A required amount of Pseudo Boehmite, triethylamine, and carbon-white were added into phosphoric acid solution under stirring conditions to form a uniform synthesis gel. The crystallization was conducted at 190 °C for 24 h in a Teflon-lined stainless steel autoclave under rotation conditions (15 rpm).

The synthesis procedures of SUZ-4 were as follows [2]: a required amount of potassium hydroxide, sodium aluminate, and tetraethylammonium hydroxide were dissolved in distilled water under stirring conditions, then silica sol was added dropwise under drastic stirring, which was continued for another 60 min. The obtained aluminosilicate gel with the formula 7.9KOH:1.0Al_2O_3:2.6TEAOH:21.2SiO_2:498.6H_2O was then transferred to a Teflon-lined stainless steel autoclave with a capacity of 100 ml, which was subsequently heated at 180 °C for 4 d under rotation (15 rpm).

ZSM-5 samples were synthesized with the gels prepared from sodium hydroxide, silica sol, sodium aluminate, tetrapropyl ammonium hydroxide, and deionized water in terms of the chemical compositions of SiO_2:xAl_2O_3:0.2TPAOH:0.3NaOH:15H_2O. The target quantity of TPAOH, $NaAlO_2$, and silica sol were dropwise added into deionized water under stirring conditions. The resultant mixture was further agitated at room temperature for 2 h to form a uniform synthesis gel. The crystallization was conducted at 170 °C for 2 d in a Teflon-lined stainless steel autoclave under rotation conditions (15 rpm). To control the zeolite particle sizes, a desired amount of silicalite-1 seed was added to the synthesis gel [3].

The synthesis procedures of MOR followed the literature with minor adjustments [4]. The sodium aluminate, sodium hydroxide, and silica sol were added into deionized water in terms of the chemical compositions of SiO_2:0.05Al_2O_3:0.3NaOH:24H_2O. The resultant mixture was further agitated at room temperature for 2 h, crystallization was subsequently carried out at 180 °C for 24 h in Teflon-lined stainless steel autoclave under rotation conditions (15 rpm).

USY and Beta were obtained from commercial manufacturers. All the as-synthesized zeolites were calcined at 550 °C for 10 h under static air to remove the organic template, followed by two rounds of ion exchange with NH_4NO_3 solution (1 M) at 80 °C. After ion exchange, the zeolites were washed with deionized water, dried at 110 °C, and then calcined under static air at 550 °C for 10 h to obtain the H-form samples.

1.2. Characterization of the catalysts

The X-ray diffraction (XRD) patterns of the catalysts were collected on a Rigaku MiniFlex II desktop X-ray diffractometer with a CuKα radiation source. The measurements were made in the 2θ range from 3° to 50° with a scanning rate of 4 ° min^{-1}.

SEM images were taken on a JSM-7001F Field emission electron microscope (JEOL Company, Tokyo, Japan).

The actual Si/Al ratios of the catalysts were determined by inductively coupled plasma atomic emission spectroscopy (ICP-AES). To prepare the solution for elemental analysis, 2 ml of hydrofluoric acid was used to dissolve 40 mg of catalyst sample; the solution was then diluted to 1000 ml with deionized water.

The nitrogen physisorption on the zeolite catalysts was performed at -195.7 °C with a physisorption analyzer (ASAP 2000, Micromeritics Instrument Co., USA). The catalyst samples were degassed under high vacuum at 200 °C for 8 h prior to the measurement. The surface area was calculated from the adsorption branch in the range of relative pressure from 0.05 to 0.25 by Brunauer–Emmett–Teller (BET) method; the pore volume was estimated at a relative pressure of 0.99.

The acid properties of the zeolites were assessed by both temperature-programmed desorption of ammonia (NH_3-TPD) and infrared spectroscopy of pyridine adsorption (Py-IR) techniques. NH_3-TPD was performed on a Micromeritics AutoChem II 2920 chemisorption analyzer. Typically, 100 mg of sample was pre-treated in argon flow (30 ml min^{-1}) at 550 °C for 2 h and then cooled down to 120 °C. The saturated adsorption of NH_3 was carried out by introducing gaseous NH_3 (5 vol% in argon, 30 ml/min) for 0.5 h. The catalyst was flushed with argon at 120 °C for 2 h to remove physisorbed NH_3 from the catalyst surface. To get the NH_3-TPD profile, the zeolite sample was then heated from 120 to 550 °C at a ramp of 10 °C min^{-1}; the amount of NH_3 released during heating for desorption was recorded by a thermal conductivity detector (TCD). The quantities of weak and strong acid sites were determined by the amounts of ammonia desorbed at 120–250 and 250–550 °C, respectively, through integrating the NH_3-TPD profile in each temperature interval. The relationship between acid amount and NH_3-TPD profile were established by measuring the change of peak area as a function of amounts of ammonia introduced into TCD before the test began.

The FT-IR spectra for pyridine adsorption (Py-IR) were measured on a Bruker Tensor 27 FT-IR spectrometer. The zeolite sample was first pressed into a self-supported wafer. Prior to the measurement, the sample cell was evacuated to 10^{-2} Pa at 450 °C for 2 h; the IR spectra were then recorded at room temperature. Next, pyridine vapor was introduced into the cell at room temperature for 1 h. After that, the zeolite samples were degassed for 2 h at 150, 250, and 350 °C and then the spectra was recorded and the background was subtracted. The concentrations of different acidic sites were calculated by following the procedures reported by Madeira et al. [5].

1.3. Other Catalytic tests

DMM decomposition and disproportionation over acid sites may lead to the formation of CH_3OH (Me), $HCOOCH_3$ (MF), and CH_3OCH_3 (DME) [6-8]. TOM decomposition may produce HCHO (FA), which is also converted to MF by the Tishchenko reaction [9]. To further understand the source of related byproducts, DMM disproportionation and TOM decomposition over zeolites were also tested.

The DMM disproportionation was conducted over HZSM-5 with a Si/Al ratio of 17 at different temperatures. For each test, 3.8 g DMM and 0.15 g HZSM-5 were loaded in a 25-ml Teflon-lined stainless steel autoclave, and the mixture was stirred continuously for 45 min. The decomposition of TOM over ZSM-5 with a Si/Al ratio of 17 and USY with a Si/Al ratio of 10 was also conducted in the same stainless steel autoclave. 10.0 g CH_2Cl_2, 1.0 g TOM, and 0.15 g HZSM-5 were loaded; the mixture was stirred continuously at 120 °C for 45 min.

1.4. The adsorption energy and electrostatic interaction of reacting molecules with the zeolite framework

The adsorption energy of DMM and TOM and the electrostatic interaction of reacting molecules with zeolite frameworks were investigated with the density functional theory (DFT) method. 49T and

78T cluster models were used to represent ZSM-5 and USY. A silicon atom is substituted with an aluminum atom to form a Brønsted acid site over ZSM-5 and USY. This acid site is located in the intersection of ZSM-5 or supercage of USY and can provide the maximal space available to accommodate various guest molecules. Terminal hydrogen atoms are used to saturate the peripheral silicon atom in the cluster model; the distances between the hydrogen atoms and the corresponding silicon atoms are 1.47 Å, and the direction of Si–H bonds is along the pre-existing Si–O bonds. All density functional theory (DFT) calculations were performed with the Gaussian 09 package [10]. The ωB97X-D functional and the 6-31G (d, p) basis set were used in all geometry optimizations and frequency calculations of all cluster models. The 14T active region and the reacting molecules were allowed to relax, while the rest of the structure was fixed at the crystallographic coordinates during geometry optimizations. To obtain high accurate interaction energies, single-point calculations with the 6-311+G (2df, 2p) basis set were refined by the ωB97X-D functional. To visualize the noncovalent interactions in zeolite systems, the isosurface plots of the reduced density gradient (RDG) for some intermediates confined in H-ZSM-5 were obtained by calculating the RDG functions and quantity sign ($\lambda 2$)ϱ with the Multiwfn software[11]. The calculations for other zeolites were also carried out in a similar way.

1.5. The detailed analysis procedures by gas chromatography

The products after reaction, including $PODE_n$, MeOH, FA, MF, DME and unreacted reactants, were measured with decane as an internal standard by two gas chromatographs: one is Shimadzu GC-2014C equipped with a FID detector and DB-1 capillary column for determining PODE*n*, DMM and TOM; the other one is Shimadzu GC-14B equipped with a TCD detector and Porapak T packed column for Methanol (MeOH), formaldehyde (FA), methyl formate (MF), dimethyl ether (DME), DMM, water, etc. Two typical GC chromatograms are showed in Figure S11.

2. Additional Results of Catalyst Characterization and Reaction Tests:

2.1. Additional catalyst characterization results

The XRD patterns in Figure S1 illustrate that the as-synthesized zeolites are well crystallized. The SEM images in Figure S2 suggest different samples show totally different crystal morphologies and all the samples are composed of uniform crystals that also corroborate the XRD results. The USY aggregates are composed of cubic crystals with an average size of approximately 1 μm. Beta particles exhibit a ball-like shape and their size is in the range of 0.3–0.5 μm. ZSM-5 particles exhibit regular spindle morphology and their average size is larger than 0.6 μm. SUZ-4 crystals exhibit a rod-like structure and the length of each rod is approximately 1 μm. SAPO-34 crystals exhibit regular cube-like shapes and the particle size is approximately 2 μm. MOR crystals exhibit accumulated plate morphology analogous to ellipsoids, and the average thickness is approximately 1 μm.

{Figure S1 & Figure S2}

Figure S3 shows Py-FTIR profiles of each zeolite at different temperatures, which were usually applied to study the nature and strength of the catalyst's acidic sites [12-14]. The two absorption peaks located at 1450 and 1540 cm^{-1} correspond to the Lewis and Brønsted acid sites, respectively. The intensity of absorption peaks at different temperatures may represent the strength of acid sites. A similar trend was observed for all the samples except SAPO-34. With increasing temperatures, the signal corresponding to Brønsted and Lewis acid sites decreased, which is attributed to desorption of parts of the pyridine molecule. In order to analyze acid properties accurately, the Py-FTIR profiles at 250 °C were selected as research subjects for comparison and quantitative analysis.

{Figure S3}

2.2. Selection of appropriate reaction conditions

The effect of the reaction time on the reactant conversion and $PODE_{2\text{-}8}$ selectivity in the synthesis of $PODE_n$ over HZSM-5 with a Si/Al molar ratio of 340 was considered. As shown in Fig. S4, when the reaction time exceeds 45 min, the conversions of DMM and TOM and mass selectivity to $PODE_{2\text{–}8}$ stay roughly the same, suggesting that a reaction time of 45 min is sufficient and appropriate for the synthesis of $PODE_n$ over HZSM-5.

{Figure S4}

The effect of the feed DMM/TOM molar ratio ranging from 1.5 to 5 on the reactant conversion and product distribution in the synthesis of $PODE_n$ over HZSM-5 with a Si/Al molar ratio of 340 was considered. As shown in Figure S5, a DMM/TOM molar ratio of 2 is appropriate to obtain high yields of $PODE_{2\text{–}8}$.

{Figure S5}

The effect of the HZSM-5 catalyst amount on the reactant conversion and product distribution in the synthesis of $PODE_n$ over HZSM-5 with a Si/Al molar ratio of 340 was considered. As shown in Fig. S6, DMM conversion decreases and TOM conversion increases gradually with the increase of catalyst usage up to 5.0 wt.%, whereas the mass selectivity to $PODE_{2\text{–}8}$ stays roughly the same. Above that, for example when the amount of catalyst used reaches 10 wt.%, the mass selectivity to $PODE_{3\text{–}8}$ decreases, suggesting that an excessive amount of acid catalyst may promote the decomposition of long-chain $PODE_n$ products. As a result, the optimized amount of catalyst used in the reaction is about 5 wt.%, which gives the highest yield of $PODE_{2\text{–}8}$.

{Figure S6}

A series of ZSM-5 zeolites with different particle sizes were synthesized through a seed-assisted organic template route. The SEM images in Figure S7 (Ⅰ) suggest that all the samples are composed of uniform crystals and that particle sizes are between 200 nm and 2000 nm. The effect of particle size on the reactant conversion and product distribution in the synthesis of $PODE_n$ over HZSM-5 with a Si/Al molar ratio of 340 was also considered. As shown in Fig. S4, the conversions of DMM and TOM and mass selectivity to $PODE_{2\text{–}8}$ stay roughly the same over ZSM-5 with different particle sizes, suggesting that the zeolite particle size has little effect on the synthesis of $PODE_n$.

{Figure S7}

2.3. DMM disproportionation over H-ZSM-5

DMM disproportionation over H-ZSM-5 with a Si/Al ratio of 17 at different temperatures was tested and the results were shown in Figure S8. It can be seen that $PODE_2$ and FA are also detected, except MeOH, MF, and DME, suggesting that only DMM disproportionation over zeolite acid sites produces small amounts of $PODE_2$ under certain conditions.

{Figure S8}

2.4. The adsorption energy and electrostatic interaction of reacting molecules with SUZ-4,Beta and MOR

{Figure S9 & Table S1}

2.5. TOM decomposition over ZSM-5 and USY

Figure S9 shows TOM conversion and product selectivity for the decomposition of TOM alone over ZSM-5 and USY at 120 °C. At 120 °C, MF and FA are the primary products over ZSM-5 and USY, respectively. The results suggest that the intermediate species FA over ZSM-5 can be converted to MF preferentially at high temperatures, whereas the intermediate species FA over USY can take part in the chain propagation forming $PODE_n$, which becomes the dominant product.

{Figure S10}

2.6. Comparison of HZSM-5(Si/Al=17) and some recently reported catalysts for the synthesis of PODE$_n$[15-20].

{Table S2}

References

1. Li J. F.; Fan W. B.; Dong M.; He Y.; Qin Z. F.; Wang J. G. Chem. Synthesis and MTO Catalytic Performance of SAPO-34. J.Chinese Universities. 2011,32(3), 765—771.
2. Gao S. ; Wang X. P. ; Wang X. Z. ; Bai Y. X. Green synthesis of SUZ-4 zeolite controllable inmorphology and SiO_2/Al_2O_3 ratio. Micropor. Mesopor. Mat. 2013,174, 108–116.
3. Xu F.; Dong M.; Gou W.; Li J.; Fan W.; Qin Z.; Wang J. Rapid tuning of ZSM-5 crystal size by using polyethylene glycol or colloidal silicalite-1 seed. Micropor. Mesopor. Mat. 2012,163,192-200.
4. Kim G. J.; Ahn W. S. Direct synthesis and characterization of high-SiO_2-content mordenites. Zeolites, 1991, 11, 745–750.
5. Madeira F.; Tayeb K.; Pinard L.; Vezin H.; Maury S.; Cadran N. Ethanol transformation into hydrocarbons on ZSM-5 zeolites: Influence of Si/Al ratio on catalytic performances and deactivation rate. Study of the radical species role. Appl. Catal. A, 2012, 443–444, 171–180.
6. Shapovalov V.; Bell A. T. Theoretical Study of Zeolite-Catalyzed Dimethoxymethane Carbonylation to Methyl Methoxyacetate. J. Phys. Chem. C 2010, 114, 17753–17760.
7. Celik F. E.; Kim T.; Mlinar A. N.; Bell A. T. An investigation into the mechanism and kinetics of dimethoxymethane carbonylation over FAU and MFI zeolites. J. Catal. 2010, 274, 150–162.
8. Celik F. E.; Kim T.; Bell A. T. Effect of zeolite framework type and Si/Al ratio on dimethoxymethane carbonylation. J. Catal. 2010, 270, 185-195.
9. Burger J.; Siegert M.; Ströfer E.; Hasse H. Poly(oxymethylene) dimethyl ethers as components of tailored diesel fuel: Properties, synthesis and purification concepts. Fuel, 2010, 89, 3315–3319.
10. Frisch M. J.; Trucks G. W.; Schlegel H. B.; Scuseria G. E.; Robb M. A.; Cheeseman J. R.; Scalmani G.; Barone V.; Mennucci B.; Petersson G. A.; Nakatsuji H.; Caricato M.; Li X.; Hratchian H. P.; Izmaylov A. F.; Bloino J.; Zheng G.; Sonnenberg J. L.; Hada M.; Ehara M.; Toyota K.; Fukuda R.; Hasegawa J.; Ishida M.; Nakajima T.; Honda Y.; Kitao O.; Nakai H.; Vreven T.; Montgomery J. A.; Peralta J. E.; Ogliaro F.; Bearpark M.; Heyd J. J.; Brothers E.; Kudin K. N.; Staroverov V. N.; Kobayashi R.; Normand J.; Raghavachari K.; Rendell A.; Burant J. C.; Iyengar S. S.; Tomasi J.; Cossi M.; Rega N.; Millam N. J.; Klene M.; Knox J. E.; Cross J. B.; Bakken V.; Adamo C.; Jaramillo J.; Gomperts R.; Stratmann R. E.; Yazyev O.; Austin A. J.; Cammi R.; Pomelli C.; Ochterski J. W.; Martin R. L.; Morokuma K.; Zakrzewski V. G.; Voth G. A.; Salvador P.; Dannenberg J. J.; Dapprich S.; Daniels A. D.; Farkas O.; Foresman J. B.; Ortiz J. V.; Cioslowski J. and Fox D. J. Gaussian 09, revision D.01, Gaussian, Inc., Wallingford CT, 2009.
11. Lu T. and Chen F. W. Multiwfn: A multifunctional wavefunction analyzer. J. Comput. Chem., 2012, 33, 580–592.
12. García-Trenco A.; Martínez A. Direct synthesis of DME from syngas on hybrid CuZnAl/ZSM-5 catalysts: New insights into the role of zeolite acidity. Appl. Catal. A. 2012, 411-412, 170-179.
13. Ding X.; Geng S.; Li C.; Yang C.; Wang. G. Effect of acid density of HZSM-5 on the oligomerization of ethylene in FCC dry gas. J. Nat. Gas Chem., 2009, 18, 156-160.
14. Li Y. C. ; Wang H.; Dong M.; Li J. F.; Qin Z. F.; Wang J. G.; Fan W. B. Effect of zeolite pore structure on the diffusion and catalytic behaviors in the transalkylation of toluene with 1,2,4-trimethylbenzene. RSC Adv., 2015,5, 66301–66310.
15. Wang W. F.; Gao X. J.; Yang Q.; Wang X. X.; Song F. E.; Zhang Q. D.; Han, Y. Z.; Tan Y. S.. Vanadium oxide modified H-beta zeolite for the synthesis of polyoxymethylene dimethyl ethers from dimethyl ether direct oxidation. Fuel, 2019,238 , 289–297.

16 Song H. Y.; Li R. Y.; Jin F. X.; Li Z.; Chen J. Efficient and reusable zeolite-immobilized acidic ionic liquids for the synthesis of polyoxymethylene dimethyl ethers. Molecular Catalysis. 2018,455, 179–187.

17 Gruenert A.; Losch P.; Ochoa-Hernandez C.; Schmidt W.; Schueth F. Gas-phase synthesis of oxymethylene ethers over Si-rich zeolites. Green Chem.,2018, 20,4719–4728.

18 Xue Z. Z.; Shang H. Y.; Zhang Z. L.; Xiong C. H.; Lu C. B.; An G. J. Efficient Synthesis of Polyoxymethylene Dimethyl Ethers on Al-SBA-15 Catalysts with Different Si/Al Ratios and Pore Sizes. Energy Fuels. 2017, 31, 279–286.
19 Xue Z. Z.; Shang H. Y.; Xiong C. H.; Lu C. B.; An G. J; Zhang Z. L. Cui C. T; Xu M.L. Synthesis of polyoxymethylene dimethyl ethers catalyzed by sulfonic acid-functionalized mesoporous SBA-15. RSC Adv.,2017, 7, 20300–20308.
20 Li H. J; Song H. L; Chen L. W; Xia C. G. Designed $SO_4^{2-}/Fe_2O_3-SiO_2$ solid acids for polyoxymethylene dimethyl ethers synthesis: The acid sites control and reaction pathways. Applied Catalysis B: Environ., 2015, 165, 466–476.

Captions

Figure S1. The XRD patterns of various zeolite catalysts.

Figure S2. SEM images of various zeolite catalysts. (a): USY; (b): Beta; (c): ZSM-5; (d): SUZ-4; (e): SAPO-34; (f): MOR.

Figure S3. The Py-IR profiles of each zeolite at different temperatures.

Figure S4. Effect of the reaction time on the reactant conversion and $PODE_{2\text{-}8}$ selectivity over HZSM-5 with a Si/Al molar ratio of 340.

Figure S5. Effect of the feed DMM/TOM molar ratio on the reactant conversion and $PODE_{2\text{-}8}$ selectivity over HZSM-5 with a Si/Al molar ratio of 340.

Figure S6. Effect of the HZSM-5 catalyst amount on the reactant conversion and product distribution over HZSM-5 with a Si/Al molar ratio of 340.

Figure S7. (Ⅰ) SEM images of ZSM-5 with different particle sizes. (a) 200 nm, (b) 500 nm, (c) 1 μm, (d) 2 μm. (Ⅱ) The effect of particle size on the catalytic performance over HZSM-5 with a Si/Al molar ratio of 340.

Figure S8. DMM disproportionation over H-ZSM-5 with a Si/Al ratio of 17 at different temperatures.

Figure S9. Isosurface plots of the reduced density gradient (s = 0.500 a.u.) for the coadsorption of DMM and TOM in Beta (a) and MOR (b). The isosurfaces of the reduced density gradient are colored according to the values of the quantity sign $(\lambda 2)\varrho$, as indicated by the RGB scale. VDW: van der Waals interactions.

Figure S10. TOM conversion and product selectivity for TOM decomposition over ZSM-5 and USY at 120 °C.

Figure S11. Two typical GC chromatograms from Shimadzu GC-2014C and Shimadzu GC-14B.

Table S1. Adsorption energy of DMM, TOM, and their combination in SUZ-4, Beta and MOR.

Table S2. Comparison of HZSM-5(Si/Al=17) and some recently reported catalysts for the synthesis of $PODE_n$.

Figure S1.

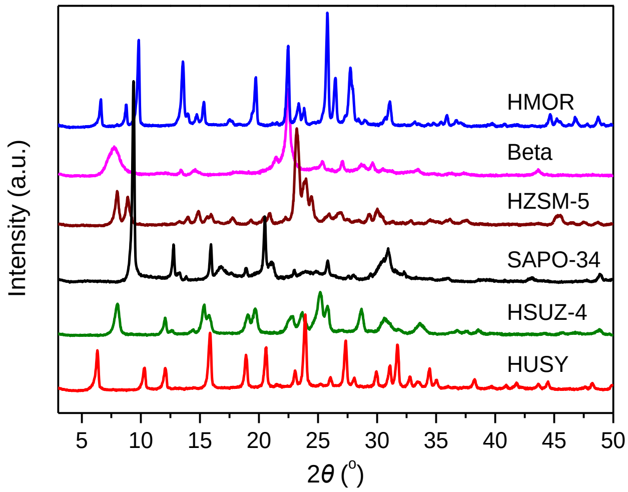

Figure S1. The XRD patterns of various zeolite catalysts.

Figure S2.

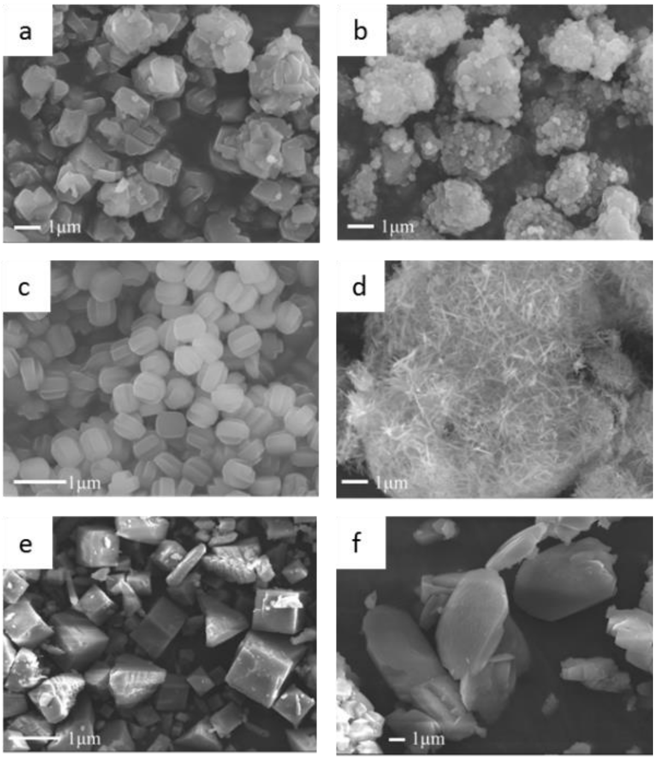

Figure S2. SEM images of various zeolite catalysts.

(a): USY; (b): Beta; (c): ZSM-5; (d): SUZ-4; (e): SAPO-34; (f): MOR.

Figure S3.

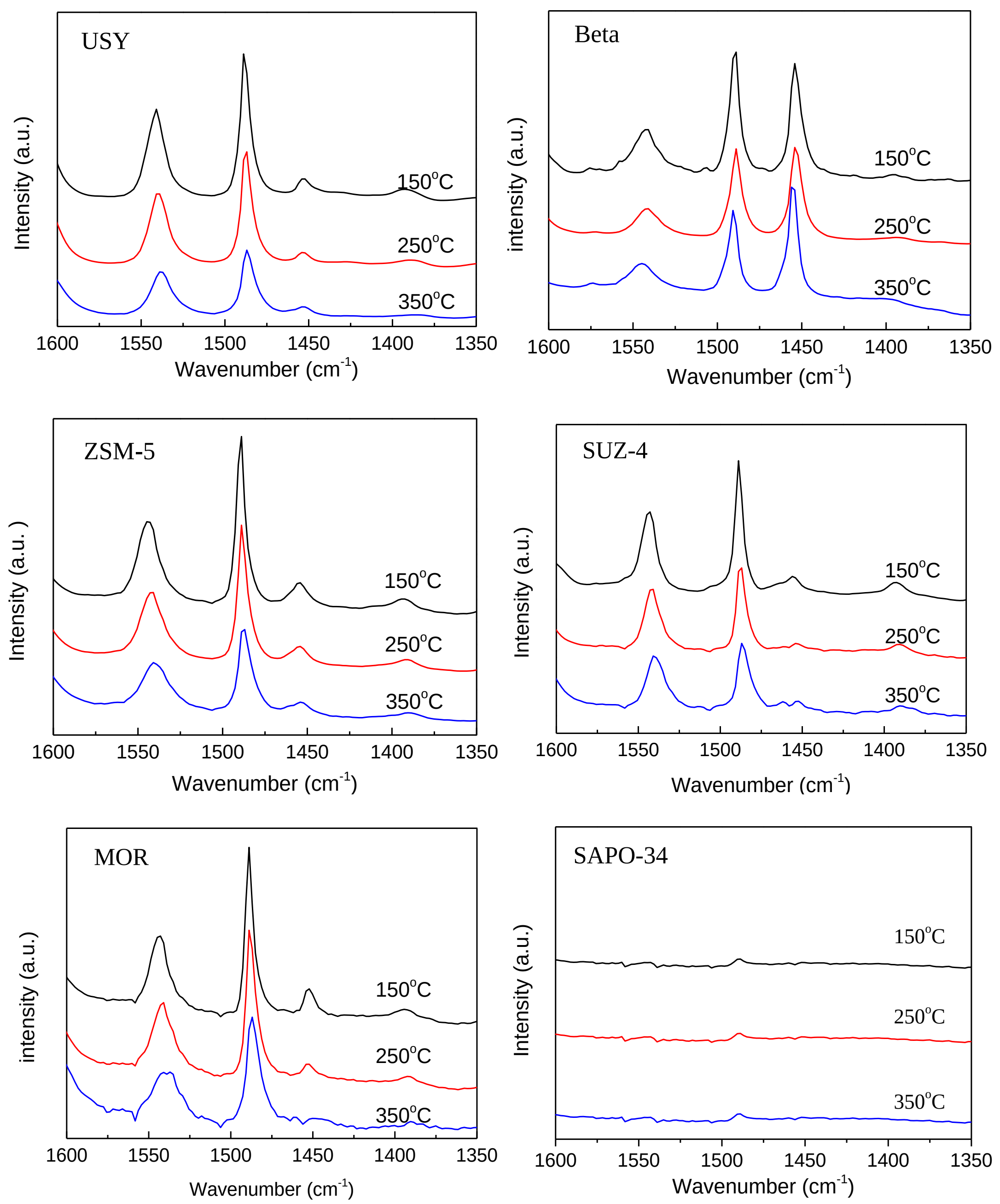

Figure S3. The Py-IR profiles of each zeolite at different temperatures.

Figure S4.

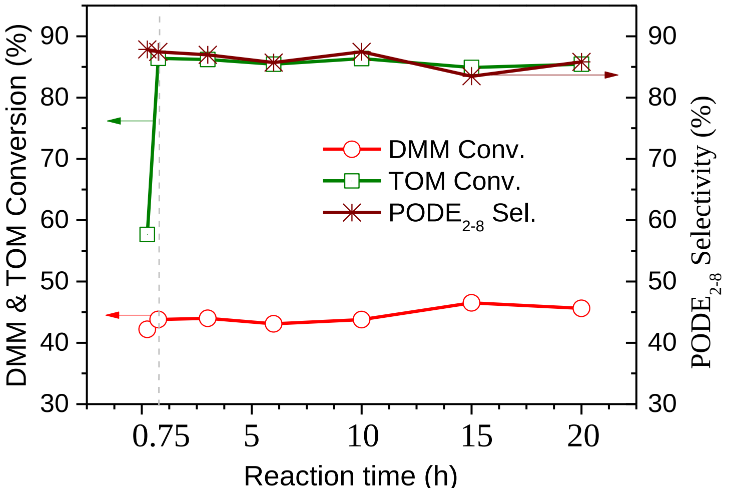

Figure S4. Effect of the reaction time on the reactant conversion and product distribution in the synthesis of PODE*n* over HZSM-5 with a Si/Al molar ratio of 340. The reaction was carried out at 120 °C, with a feed DMM/TOM molar ratio of 2 and catalyst amount of 5 wt.%

Figure S5

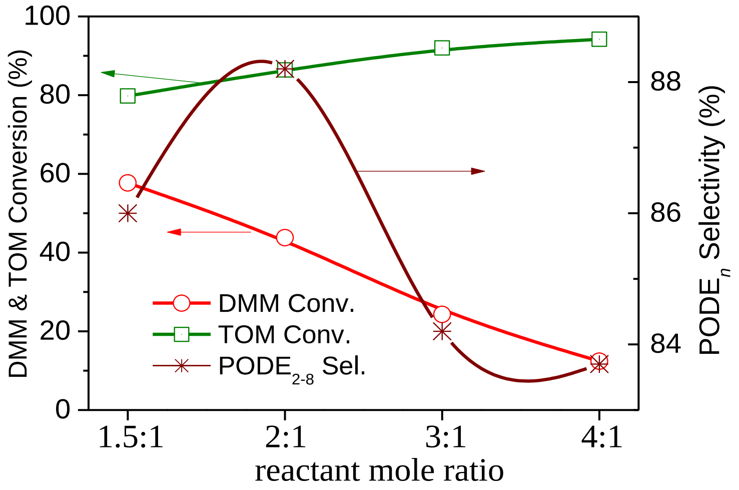

Figure S5. Effect of the feed DMM/TOM molar ratio on the reactant conversion and PODE$_{2-8}$ selectivity over HZSM-5 with a Si/Al molar ratio of 340. The reaction was carried out at 120 °C for 45 min, with a catalyst amount of 5 wt.%.

Figure S6.

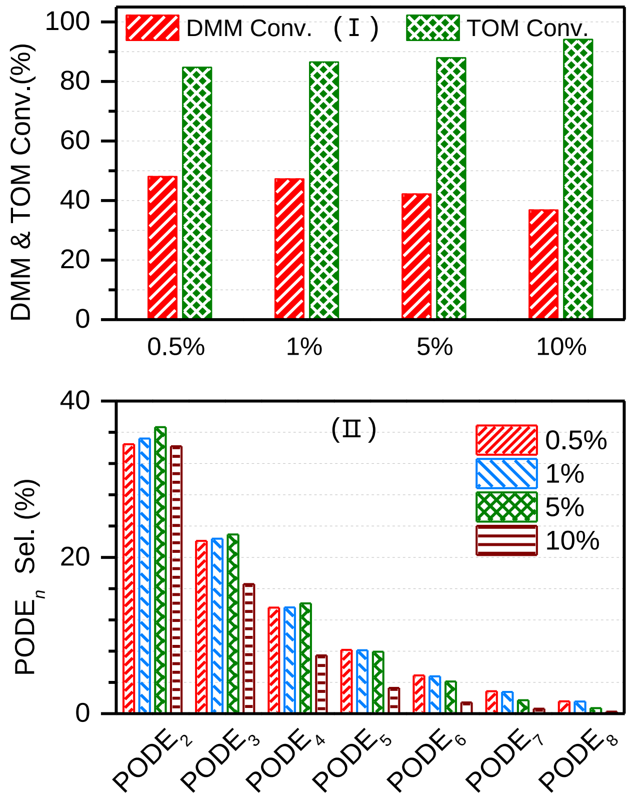

Figure S6. Effect of the HZSM-5 catalyst amount on the reactant conversion (I) and product distribution (II) in the synthesis of PODE$_n$ over HZSM-5 with a Si/Al molar ratio of 340. The reaction was carried out at 120 °C for 45 min, with a feed DMM/TOM molar ratio of 2.

Figure S7.

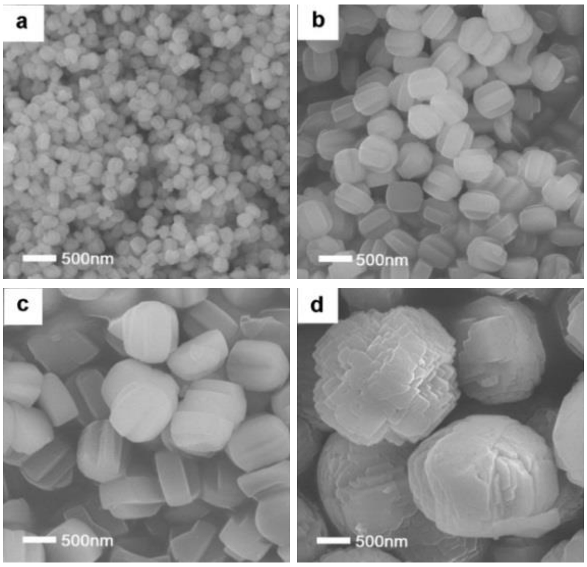

(I)

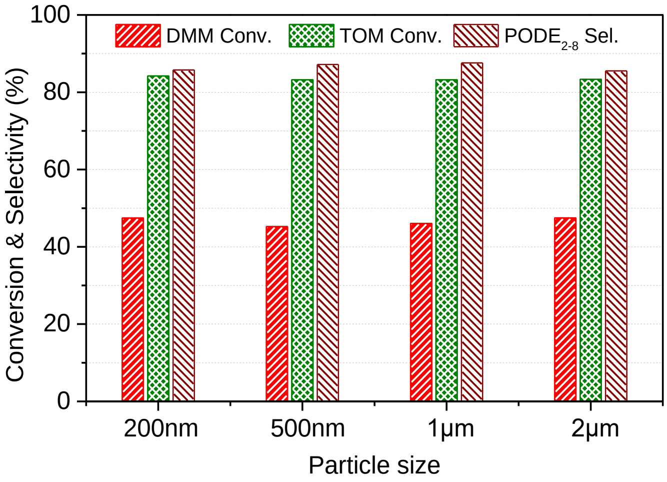

(II)

Figure S7. (I) SEM images of ZSM-5 with different particle sizes. (a) 200 nm, (b) 500 nm, (c) 1 μm, and (d) 2 μm. (II) The effect of particle size on the catalytic performance over HZSM-5 with a Si/Al molar ratio of 340. The reaction was carried out at 120 °C for 45 min, with a feed DMM/TOM molar ratio of 2 and catalyst amount of 5 wt.%

Figure S8.

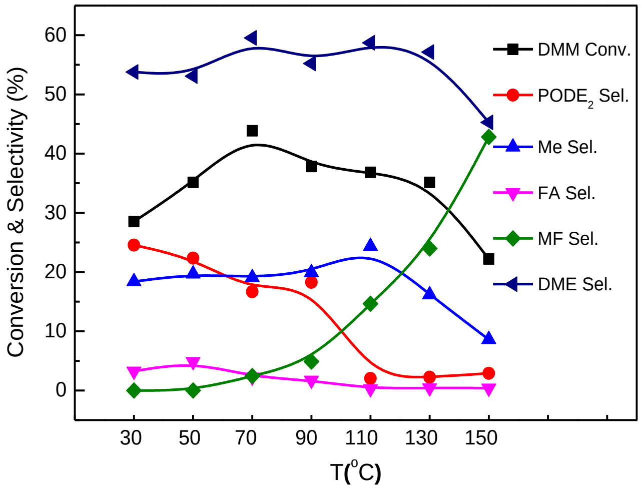

Figure S8. DMM disproportionation over H-ZSM-5 with a Si/Al ratio of 17 at different temperatures.

Figure S9.

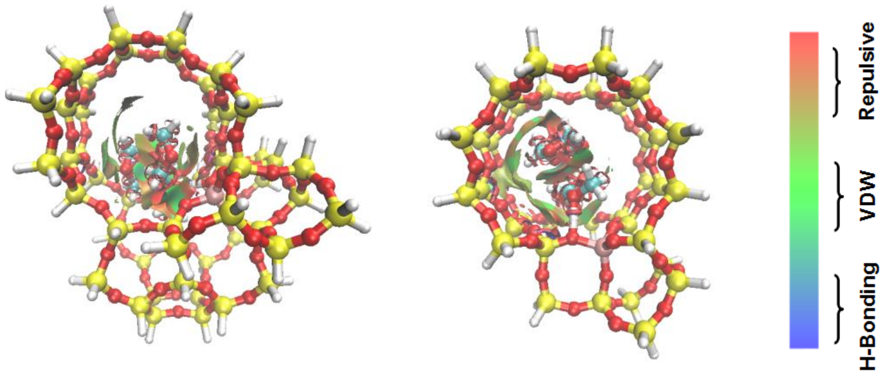

(a) **(b)**

Figure S9. Isosurface plots of the reduced density gradient (s = 0.500 a.u.) for the coadsorption of DMM and TOM in Beta (a) and MOR (b). The isosurfaces of the reduced density gradient are colored according to the values of the quantity sign ($\lambda 2$)ϱ, as indicated by the RGB scale. VDW: van der Waals interactions.

Figure S10.

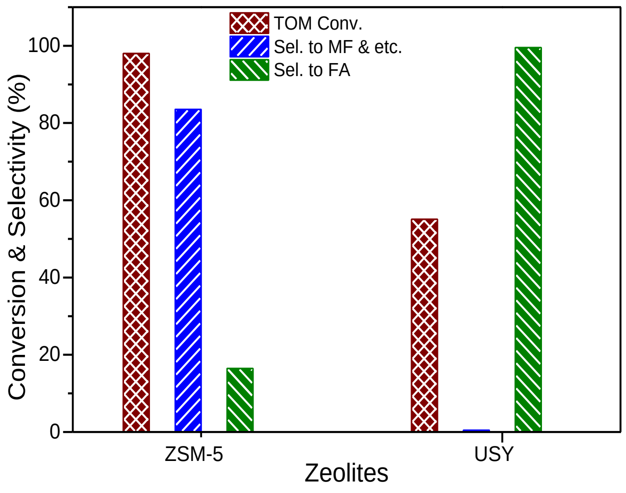

Figure S10. TOM conversion and product selectivity for TOM decomposition over ZSM-5 and USY at 120 °C.

Figure S11

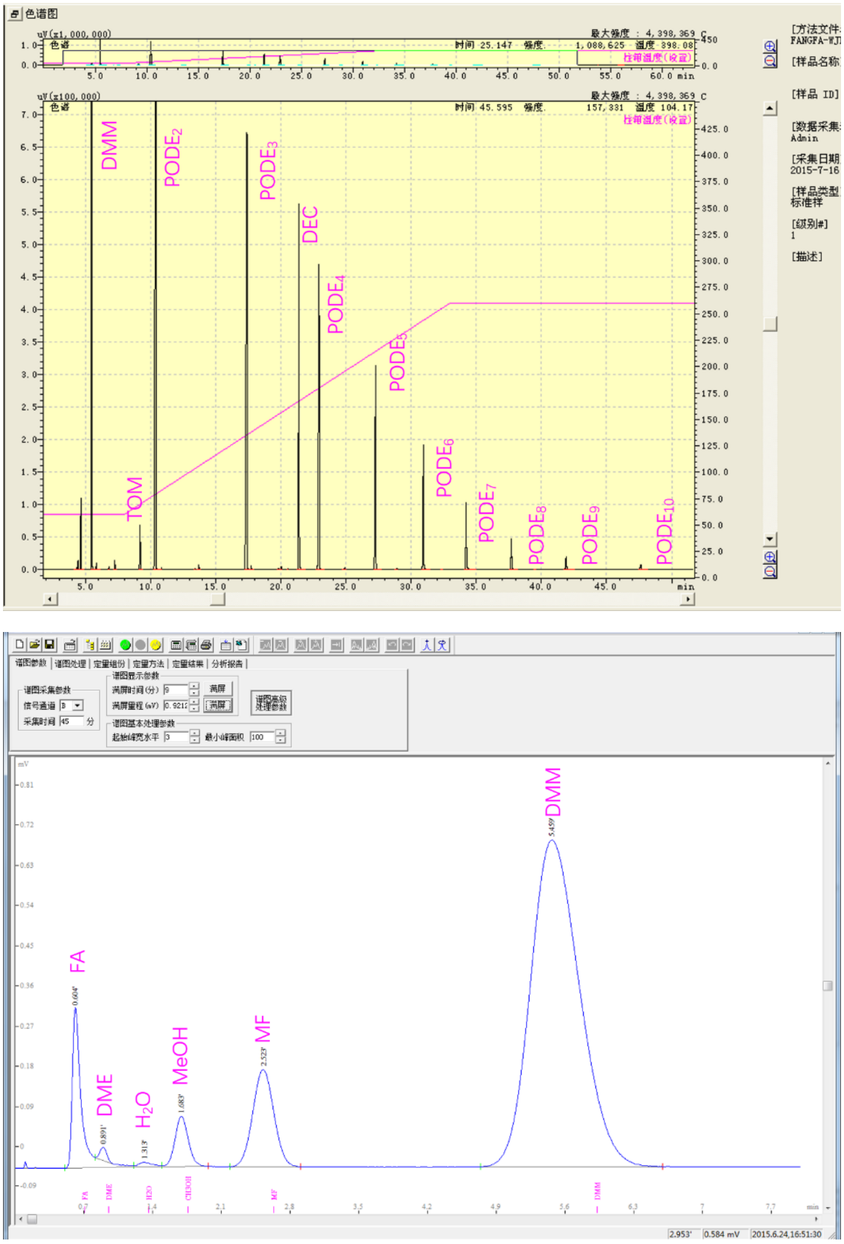

Figure S11. Two typical GC chromatograms from Shimadzu GC-2014C and Shimadzu GC-14B.

Table S1.

Table S1. Adsorption energy of DMM, TOM, and their combination in SUZ-4, Beta and MOR.

Samples	DMM (kJ/mol)	TOM (kJ/mol)	DMM+TOM (kJ/mol)
FAU	-60	-53	-107
ZSM-5	-90	-65	-117
SUZ-4	-92	-68	-121
MOR	-75	-61	-114
BEA	-78	-60	-115

Table S2.

Table S2. Comparison of HZSM-5(Si/Al=17) and some recently reported catalysts for the synthesis of $PODE_n$.

Catalysts	Reactants	Reaction conditions	Performance	References
H-ZSM-5-17	DMM<br>TOM	90°C , $n_{(DMM)/(CH2O)}$ = 2,<br>atmospheric pressure,<br>45min,<br>catalyst loading: 5%.	DMM Conv. : 53.9%<br>TOM conv. : 98.9%<br>$PODE_{2-8}$ Sel. : 85.8%	This work
V_2O_5/H-beta	DME<br>O_2	230 °C, $n_{DME/O2}$=1,<br>atmospheric pressure,<br>2h, GHSV=3600h^{-1}.	DME Conv. : 9.2%<br>$PODE_{1-3}$ Sel.: 74.3%	15
[NaZSM-5IMBs]HSO_4	DMM<br>TOM	110 °C, $n_{DMM/HCHO}$ =1.2,<br>2.5 MPa, 2 h,<br>Catalyst loading: 5 % .	TOM Conv.： 90.3%<br>$PODE_{3-8}$ Sel.:53.5%	16
H-MOR-40	MeOH<br>FA	130°C,<br>1Mpa, 40 minutes,<br>0.5 g.	Average of FA and<br>MeOH Conv. ： 49%<br>$PODE_{1-3}$ Sel.: 95%	17
Al-SBA-15	DMM<br>TOM	100 °C, $n_{(DMM)/(CH2O)}$ = 1;<br>1 MPa, 1h,<br>catalyst loading: 2%.	DMM Conv. : 48.4%<br>TOM Conv. : 92.7%<br>$PODE_{2-8}$ Sel. : 98.2%	18
SO_3H-SBA-15	DMM<br>TOM	100 °C; $n_{(DMM)/(CH2O)}$ = 1;<br>1 MPa, 1h,<br>catalyst loading: 2%.	DMM Conv. : 51.8%<br>TOM Conv. : 95.6%<br>$PODE_n$ yield : 61.8%	19
$SO_4^{2-}/Fe_2O_3-SiO_2$	MeOH<br>TOM	130 °C, $n_{MeOH/TOM}$ = 1.5,<br>atmospheric pressure,<br>2 h,<br>catalyst loading: 1.5%.	MeOH Conv. : 71.6 %<br>TOM Conv. : 30.9%<br>$PODE_{1-8}$ Sel. : 68.3%	20
